# Sialyltransferases and Neuraminidases: Potential Targets for Cancer Treatment

**DOI:** 10.3390/diseases10040114

**Published:** 2022-11-28

**Authors:** Sagorika Nag, Abhimanyu Mandal, Aryaman Joshi, Neeraj Jain, Ravi Shanker Srivastava, Sanjay Singh, Arun Khattri

**Affiliations:** 1 Department of Pharmaceutical Engineering and Technology, Indian Institute of Technology, Banaras Hindu University, Varanasi 221005, India; 2 Department of Chemical Engineering and Technology, Indian Institute of Technology, Banaras Hindu University, Varanasi 221005, India; 3 Division of Cancer Biology, CSIR-Central Drug Research Institute, Lucknow 226031, India; 4 Department of Pharmacology, Career Institute of Medical Sciences & Hospital, Lucknow 226020, India

**Keywords:** sialyltransferases, neuraminidases, ovarian cancer, breast cancer, pancreatic cancer, chemoresistance, inhibitors

## Abstract

Cancers are the leading cause of death, causing around 10 million deaths annually by 2020. The most common cancers are those affecting the breast, lungs, colon, and rectum. However, it has been noted that cancer metastasis is more lethal than just cancer incidence and accounts for more than 90% of cancer deaths. Thus, early detection and prevention of cancer metastasis have the capability to save millions of lives. Finding novel biomarkers and targets for screening, determination of prognosis, targeted therapies, etc., are ways of doing so. In this review, we propose various sialyltransferases and neuraminidases as potential therapeutic targets for the treatment of the most common cancers, along with a few rare ones, on the basis of existing experimental and in silico data. This compilation of available cancer studies aiming at sialyltransferases and neuraminidases will serve as a guide for scientists and researchers working on possible targets for various cancers and will also provide data about the existing drugs which inhibit the action of these enzymes.

## 1. Introduction 

With the globally rising cases of various kinds of cancer, finding biomarkers or targets has become of utmost importance for proper diagnosis and treatment. According to a study conducted in the US, the number of cancer incidence cases is to be increased by 50% by 2050, and hence there needs to be a greater emphasis on the development of alternative therapies [1] Though the existing cancer treatments such as chemotherapy, immunotherapy, and early detection and prevention strategies have majorly contributed to decreasing death rates, there is still a large cohort of patients who do not respond to such strategies. A major proportion of cancer-related deaths is caused due to metastasis instead of the primary tumor, and hence stopping any event out of adhesion, communication, and migration of tumor cells serves as a possible treatment for cancer [2]. Though insights into the invasion and migration of tumor cells have been gained, there is a need to understand the genomic basis of these processes, especially the role of glycosylation in metastasis [3]. Glycosylation is a post-translational modification that takes place in the Endoplasmic Reticulum (ER) and Golgi apparatus by which various kinds of glycans are attached to peptide backbones [4]. Out of the different kinds of glycosylation processes occurring, sialylation is of critical importance because of its involvement in oncogenesis, immune response, embryonic development, etc. Sialylation involves the addition of sialic acids, bridging molecules of sugars found in the distal ends of the glycan to the glycoproteins [5]. Aberrant glycosylation and, in particular, aberrant sialylation is an established hallmark of various kinds of cancer, and studies around the same have widely suggested that enzymes involved in the regulation of sialylation can be used as potential biomarkers and targets for the treatment of cancer [6]. There are two sets of enzymes: neuraminidases and sialyltransferases, which remove and add sialic acids to glycans, respectively, thereby dynamically regulating cell surface sialylation [3]. Hypersialylation can be a result of the upregulation of sialyltransferases or downregulation of neuraminidases, or both, which leads to the accumulation of excess negatively charged sialic acids on the cell surface, further resulting in immune evasion and promotion of tumor metastasis. This also has been found to contribute to the reduced efficacy of existing cancer therapies such as radiotherapy and chemotherapy [6]. Humans have 20 subtypes of membrane-bound sialyltransferases which mostly reside in the medial and trans cisternae of the Golgi apparatus and catalyze the linkage between a carbon of sialic acid from the cytidine monophosphate N-acetylneuraminic acid (CMP-Neu5Ac) donor and hydroxyl of the glycan acceptor. However, some STs have been found in the trans-Golgi network, with two of them being expressed as post-Golgi-secreted enzymes [7]. The names of sialyltransferases are based on the carbon to which the hydroxyl group is attached, along with the type of acceptor sugar [3]. The last two decades have seen a great increase in the number of studies being conducted to analyze the role of sialyltransferases and neuraminidases in various kinds of cancer, including ovarian cancer, melanoma, breast cancer, etc. This review systematically introduces the concepts of the sialylation role of sialyltransferases and neuraminidases in cancer and summarizes studies conducted to date on the same. Though there are various reviews available that emphasize the role of sialyltransferases or particular neuraminidases in cancer progression, none of them include both sialyltransferases and neuraminidases in their study. This review fills in the existing gap and also reports all existing studies in a cancer-wise manner, which aids researchers working on finding alternate and efficient treatments for different cancers and proposes potential sialyltransferases and neuraminidases as targets for further studies.

## 2. Sialyltransferases and Neuraminidases: Types and Functions

The enzymes sialyltransferases and neuraminidases (or sialidases), which transfer and remove sialic acid residues from the terminal positions of glycoconjugates, respectively, are essential for sialic acid metabolism. Sialic acids mediate cell–cell recognition, communication, aggregation, development, carbohydrate–protein interactions, controlling the lifetimes of glycoconjugates in organisms, mediating bacterial and viral infections, tumor growth, and metastasis, with a role in immunology, microbiome, cell signaling, reproduction, and biology of the nervous system [8]. Additionally, it promotes tumor growth, inhibits cells from going through apoptosis, grows tumors, aids immune evasion, and promotes cancer detachment. The following sections describe in detail the different families of sialyltransferases and neuraminidases.

### 2.1. Types of Sialyltransferases

**Figure 1:**
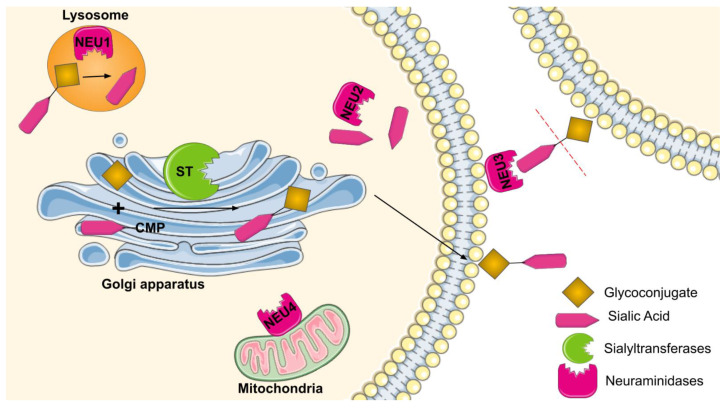
Sialylation and desialylation in the human cell. The diagram represents how the Golgi resident sialyltransferases attach sialic acids to the glycoconjugates, where at the same time, neuraminidases present in lysosomes, plasma membrane, and mitochondria act on the sialylated glycans and remove the sialic acids.

Sialyltransferases (STs) are categorized under inverting glycosyltransferases, i.e., enzymes which invert the stereochemistry of the donor’s anomeric bond (α → β). In the Golgi apparatus, these enzymes catalyze the transfer of sialic acid moiety from an activated sugar nucleotide donor, that is, CMP-Neu5Ac (Cytosine 5′-monophosphate N-acetylneuraminic acid) to non-reducing positions of acceptors such as galactose, N-Acetyl galactosamine, and other sialic acid residues (Figure 1). Till now, twenty different human STs have been identified, which are classified into four categories according to the nature of the substrates and type of linkage, namely, ST3Gal, ST6Gal, ST6GalNAc, and ST8Sia. These four classes are known to produce four different types of glycosidic bonds, namely, Neu5Acα2-6Gal, Neu5Acα2-3Gal, Neu5Acα2-6GalNAc, and Neu5Acα2-8Neu5Ac. The four subclasses of STs have been described in detail in Table 1. On the basis of commonly used names, substrate carriers (or glycan specificities), and structure (linkage formed) formed as a result of the action of the enzyme.

### 2.2. Types of Neuraminidases

Neuraminidases or sialidases are glycohydrolytic enzymes that cleave sialic acid upon nascent virion release from the cell [9]. Broadly NEUs are categorized into viral neuraminidases, bacterial neuraminidases, and mammalian neuraminidases. Mammalian neuraminidases are a family of glycoside hydrolase enzymes that catalyze the cleaving of terminal α-glycosidically linked sialic acid moiety from the surface of cells (Figure 1). In the human genome, four neuraminidases (NEU1, NEU2, NEU3, and NEU4) have been successfully identified. These four enzymes of NEU have been described in detail in Table 2. On the basis of commonly used names, substrate carriers (or glycan specificities), tissue expression, and intracellular localization. 

## 3. Role of Sialyltransferases and Neuraminidases in Tumor Growth and Metastasis

**Figure 2:**
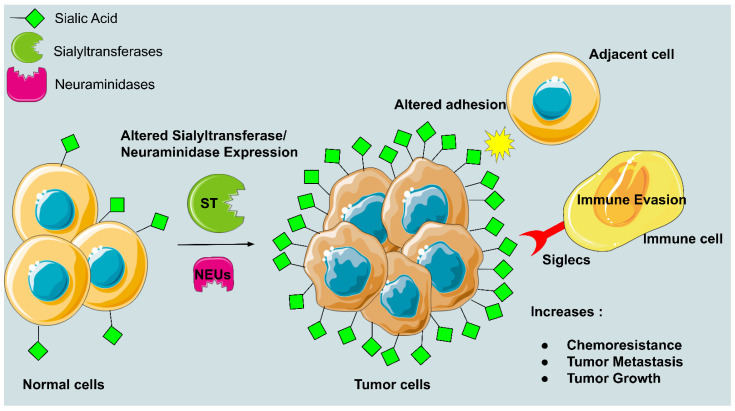
Hypersialylation in tumor cells and its adverse effects. The figure demonstrates aberrant sialylation by upregulation of sialyltransferases or downregulation of neuraminidases, or both contribute to the accumulation of excess negative charge in cancer cells leading to immune evasion, altered adhesion between adjacent cells, etc.

The enzymes known as sialyltransferases are in charge of adding sialic acid to developing glycoconjugate chains. A known feature of various malignancies, including lung, breast, ovarian, pancreatic, and prostate cancer, is the upregulation of this enzyme by 40–60%. Through several mechanisms, such as improving immune evasion and tumor cell survival and promoting tumor invasion and migration, hypersialylation aids in the spread of tumors. A surplus of the negatively charged sialic acid is present on the cell surface as a result of hypersialylation (Figure 2), which can be caused by overexpression of sialyltransferases, a downregulation of neuraminidases, or a combination of the two [12]. Through recognition by sialic acid-binding proteins, sialyltransferases play a role in the manufacture of tumor-associated sialoglycans, which in turn affect tumor development and the host’s immunological response. In this review, we go over new information about the precise processes by which altered sialylation encourages cancer metastasis during the stages of invasion, intravasation, circulation, extravasation, and colonization. 

### 3.1. Maintaining Tumor Growth and Proliferation

The capacity to multiply even in the absence of proliferative stimulation is a crucial characteristic of cancer cells. The molecular relationship between increased sialylation and cellular proliferation may only be present in some types of cancer, although it has been demonstrated that sialylation affects proliferative signaling pathways.

The discovery that ST3GAL1 overexpression promotes mammary carcinogenesis was one of the first indications that the ST3GAL family is implicated in cancer, albeit the mechanism by which this ST exerts its oncogenic activity has not been clarified [13]. The ST6GAL family has been linked to controlling the proliferation of cancer cells. Through suppression of the PI3K/AKT/GSK-3/-catenin pathway, ST6GAL1 silencing reduced the capacity of PC-3 and DU145 prostate cancer cell lines to proliferate and form colonies. Similar to this, ST6GAL2 silencing reduced the proliferation of MCF-7 and T47D breast cancer cells by stopping cell cycle progression at the G0/G1 phase and decreasing the proportion of cells in the S phase [14]. The PI3K/AKT/NF-B pathway is inhibited by ST6GALNAC1 silencing, which decreases the proliferation and clonogenicity of mouse hepatocarcinoma cell lines [15]. Similarly, ST8SIA4 knockdown inhibited MDA-MB-231 cell proliferation in vitro and tumor development in vivo, as seen by the decrease in Ki67-positive cells in the tumor tissue [16]. When ST6GALNAC5 was overexpressed in U373 MG cells, it prevented the formation of gliomas in vivo. Glioma cells have very low levels of ST6GALNAC5 expression [17]. It is interesting to note that sialyltransferase expression increases tend to enhance pro-tumorigenic and pro-metastatic effects in most cancer types, although they can have the opposite effects in malignancies arising from brain tissues, such as glioma. This might be because adult brain plasticity and regeneration depend heavily on polysialylation [18].

### 3.2. EMT Inducing Events, Invasion, and Metastasis Activating Events

An invasive phenotype of cancer requires the capacity of cancer cells to penetrate and disseminate. A number of steps are involved in this process, beginning with the local invasion of cancer cells into the surrounding tissues, then intravasation, survival in the milieu of the circulatory system, extravasation, and proliferation in the new tissue [19]. It is well known that STs actively encourage the development of invasive and metastatic characteristics in many different forms of cancer [3]. The epithelial-mesenchymal transition (EMT), a reversible cellular phenotypic switching process defined by the loss of epithelial markers in favor of a migratory mesenchymal state, has been linked to changes in sialylation. The primary catalyst for malignant tumor metastasis is EMT [20]. 

It is generally known that ST3GAL is associated with enhanced invasion and metastasis. The metastatic HCC cell line HCCLM3 has been found to migrate and invade when ST3GAL1 is expressed, and its expression is linked to a poor outcome in human HCC [21]. Additionally, through EGFR signaling, ST3GAL1 overexpression can encourage the migratory and peritoneal spread of ovarian cancer cells [22]. A further study revealed that ST3GAL1 is essential for the TGF-1-induced EMT in ovarian cancer. When ovarian cancer cells were treated with TGF-, ST3GAL1 was more highly expressed, which decreased the levels of E-cadherin and increased the levels of N-cadherin and vimentin [23]. It has been demonstrated that other ST3GAL family members control invasion and migration. For instance, ST3GAL3 altered the adhesion and invasion of breast cancer cells by upregulating the expression of molecules involved in invasion mechanisms, such as one integrin, matrix metalloproteinase (MMP)-2, MMP-9, and COX-2 [24]. It has been demonstrated that ST3GAL3 and ST3GAL4 increase the adhesion, motility, and migration of pancreatic cancer cells in vitro and the potential for metastasis in vivo [25]. The migration, invasion, and E-selectin-dependent adhesion of pancreatic cancer cells were consistently reduced when ST3GAL3 or ST3GAL4 expression was downregulated [26].

### 3.3. Immunological Evasion

For cancer cells to effectively metastasize and spread throughout the body, they must be able to evade recognition and elimination by the immune system. It has long been understood that cancer cells’ abnormal glycosylation shields them from the immune system’s devastation [27]. Particularly, sialic acid on the surface of cancer cells is thought to be critical for immune regulation and tumor immune evasion, and sialic acid inhibition may offer a therapeutic strategy to develop an immune-permissive tumor microenvironment [28]. Knocking down the sialic acid transporter results in a reduction in sialic acid in murine B16 melanoma cells. Slc35A1 has been demonstrated to improve effector T cell response, decrease the amount of 2,6-linked sialic acids on the cell surface, and boost the inflow and activity of natural killer (NK) cells, strengthening anti-tumor immunity and slowing tumor progression [29].

It has only recently been clear how certain STs contribute to the immune evasion of cancer cells. For example, ST6GAL1 has been demonstrated to enhance immune evasion in hepatocarcinoma cells by increasing levels of CD147, MMP9, MMP2, and MMP7 and suppressing T-cell proliferation [30].

### 3.4. Evading Cell Death and Apoptosis

Cancer cells must avoid and overcome cellular death in order to grow and thrive in the tumor microenvironment. Several different cell types’ programmed cell death has been linked to sialylation. The TNF family of death receptors (TNFRs) includes TNFR1, DR4, DR5, and Fas (CD95), which regulate programmed cell death. These molecules are frequently altered in human cancers, and they have been firmly linked to tumor cell survival [31]. Through ST6Gal-I-mediated hypersialylation of the Fas receptor, cancer cells are able to evade apoptosis and cell death signaling (FasR). This, in turn, prevents the internalization of Fas and the development of the death-inducing signaling complex (DISC), which disables the signaling that causes apoptosis [32]. By sialylating TNF receptor 1, internalization is hindered, and apoptosis is prevented. ST6GAL1 overexpression has a comparable effect on Tumor Necrosis Factor (TNF)- induced cell death [33]. In a study, it was discovered that the production of normal extended O-glycans could increase sensitivity to TRAIL through O-glycosylated DR4 or DR5. This is the first molecular mechanistic understanding of how tumor cell survival is aided by the expression of shortened O-glycans, such as Tn/sTn antigens [34].

## 4. Role of Sialyltransferases and Neuraminidases in Various Cancers

**Figure 3:**
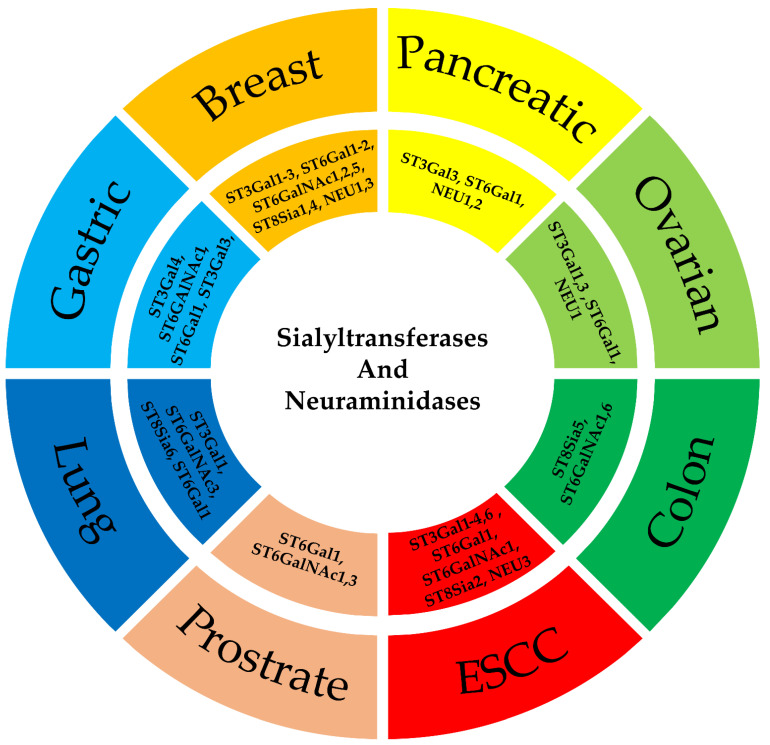
Role of sialyltransferases and neuraminidases in various cancer.

In the previous section, we saw existing research data supporting how various enzymes involved in sialylation are responsible for promoting cancer proliferation and metastasis via different mechanisms. This provides us a basis to understand how by inhibiting or overexpressing some of these particular enzymes, we might aid in the treatment of cancer. However, since here we are dealing with a total of 24 enzymes studying all of them together for multiple cancers might become complicated. Hence, we compile all relevant research studies conducted to date in a cancer-wise manner allowing one to know which of these enzymes might be the probable targets/biomarkers for different cancers (Figure 3).

### 4.1. Breast Cancer

The most common cancer detected in women with distant metastasis and poor prognosis is breast cancer. Studies have found it to be the second largest cause of death amongst female cancer patients [35]. A study conducted in 1998 reported an increase in the mRNA expression of ST3GAL2 in breast cancer stem cells is linked to increased gangliosides expression. A high expression of ST3Gal3 was found to be positively correlated with the number of axillary lymph nodes and reduced patient overall survival [36]. MUC1 is a membrane protein expressed on the luminal surface of simple epithelial cells. It has a large Extracellular Domain (ECD) consisting of repeated amino acids, each comprising five potential sites for O-glycosylation. In normal breast epithelial cells, the glycosylate units of MUC1 are transformed from core1 to core2 glycans, followed by adding polylactosamine units. However, this conversion is reduced in breast cancer cells, resulting in shorter O-glycan chains with increased sialic acid content. In a study, the staining intensity of Maakia amurensis lectin (2,3 linked sialic acid) and ST3Gal I expression was shown to be strongly positively correlated (*p* = 0.0015), demonstrating a direct linkage between an increase in sialic acid structures and ST3Gal1 mRNA expression [37]. It has also been determined that there is a strong association between ST3Gal3 expression and SLeX synthesis levels, which controls the ability of cells to adhere to rh-E selectin. This resulted in the conclusion that ST3Gal3 overexpression increases cell migration and invasiveness (*p* = 0.05) [24]. In TNBC patients, a different study found a positive correlation between high ST6GAL1 mRNA expression and a favorable relapse-free survival (RFS) rate [38]. Furthermore, knocking down ST6GAL1 with shRNA targeted to the various ST6GAL1 sites significantly reduced the aggressive breast cancer cells’ capacity to proliferate and invade. ST6GAL2 expression is associated with poor prognosis for patients [14]. Moreover, silencing ST6GAL2 in breast cancer cells reduced xenograft tumor growth in vivo [14]. Along with its presence throughout the Golgi stacks in the transfected cells, ST6GalNAc1 is seen to play a role in the core1/core2 pathway (described above) [39]. A subsequent result also established a correlation between the expression of hST6GalNAc-I and STn and concluded the expression of STn in breast cancer cells transfected with the enzyme. Additional research validated the role of ST6GalNAc2 as a novel metastasis suppressor by correlating its high expression level with increased patient survival [40] and reveals the overexpression of ST6GalNac5 leads to a decreased adhesion [41]. TNBC patients showed a connection between increased STSia8 expression and poor overall survival (OS) and disease-free survival (DFS) in a different study [42]. While there was no statistically significant difference in ER-negative people, increased ST8Sia1 mRNA expression was associated with improved disease-free survival [43]. Additionally, ST8Sia1 was silenced to limit tumor growth in a xenograft model, and ST8Sia1 was downregulated by triptolide to suppress tumor growth and lengthen survival [44]. Finally, it has been discovered that ST8Sia4 is overexpressed and aids in the development of breast cancer [16].

Suppression of NEU1 demonstrates the correlation with a decrease in cell proliferation and apoptosis enhancement by the activation of caspase3 [45]. NEU3 silencing caused apoptosis without specific stimuli [46].

### 4.2. Pancreatic Cancer

PDAC (pancreatic ductal adenocarcinoma) has a poor 5-year survival rate of fewer than 9 percent, making it one of the fatal cancers. According to recent research, ST6Gal-I, a glycosyltransferase, supports CSC (cancer stem cells) traits and functions as a survival factor to shield cells from cytotoxic assaults such as chemotherapy, radiation, and serum deprivation, and hypoxia [47,48,49,50]. In many malignancies, including ovarian, pancreatic, and colon tumors, ST6Gal-I is elevated, and high production of this enzyme is associated with a bad prognosis for the patient [51,52,53]. Pancreatic Intraepithelial Neoplasia (PanIN) has significant ST6Gal-I expression, but normal pancreatic acinar cells do not exhibit ST6Gal-I protein expression [51].

EGFR is a key factor in driving EMT, and earlier research has shown that sialylation of EGFR by ST6Gal-I encourages both basal and ligand-dependent EGFR activation in pancreatic and ovarian cancer cells [54]. The EGFR inhibitor, Erlotinib, has been approved by the US Food and Drug Administration to treat PDAC in combination with gemcitabine since EGFR is elevated in about 85% of patients with PDAC [55]. According to research, which showed that EGFR activation in various cancer cell lines was α2-6 sialylation-dependent, ST6Gal-I OE in Suit2 cells increased α2-6 sialylation and basal EGFR activation, whereas ST6Gal-I KD in S2-013 and S2-LM7AA decreased EGFR sialylation and activation. Notably, erlotinib treatment removed sialylation-dependent variations in EGFR activation and reversed the effects of ST6Gal-I on the expression of EMT markers and cell invasiveness in the Suit2 and S2-LM7AA lines. These findings provide compelling evidence for the idea that sialylated EGFR participates in the EMT-promoting effect of ST6Gal-I [56].

Studies have shown that greater 2,3- and 2,6-linked sialic acids (SAs) are found in pancreatic cancer cells, which most contribute to the disease’s higher risk of metastasis [57]. Lysosomal (Neu1), cytosolic (Neu2), membrane-bound (Neu3), and luminal (Neu4) sialidases are the four sialidases found in mammalian cells. They differ in their enzymatic properties and substrate specificity. They act differently throughout carcinogenesis and are crucial for the equilibrium of sialylation [46,58]. After comparing the status of Neu1/Neu2/Neu3/Neu4 in cancer and then in normal tissue specimens, it was concluded that the loss of Neu2 may aid in greater sialylation status in pancreatic cancer manifestation [59]. According to several findings, greater sialylation status influences the ST3GalIII and 2,3 sialyltransferase galactosyl transferases in PDAC, which in turn affects cell motility, adhesion, and metastasis [60]. In response to apoptotic triggers such as serum deprivation, which drastically decreased the growth rate, Neu2 overexpression displayed apoptosis vulnerability. More importantly, this event affected crucial cell cycle regulatory molecules, increased the expression of pro-apoptotic and decreased the expression of anti-apoptotic proteins, pushing Neu2-overexpressed cells toward apoptosis as indicated by an increased number of cells in the late apoptotic stage. It is interesting to note that in pancreatic cancer, α2,3linked SAs are thought to be the primary substrate for Neu2 [59].

### 4.3. Ovarian Cancer

Ovarian cancer is amongst the most common cancers affecting females around the world and is accompanied by an extremely poor prognosis and significantly higher death rates [61]. A large number of studies suggest that targeting the sialyltransferases and neuraminidases might be a potential strategy to prevent metastasis in this particular cancer. One such study involving qRT-PCR, western blotting, and immunohistochemistry assessed the expression of sialyltransferase ST3GAL1 in ovarian cancer tissue and cell lines and found it to be upregulated. Their results also indicated that overexpressing ST3GAL1 promoted cell growth, migration, and invasion in ovarian cancer cells, whereas inhibiting STG3GAL1 expression had the opposite results. They also investigate ST3GAL1′s participation in EMT via various studies and conclude that it can influence the level of cell migration and invasion induced by TGF-β1 [23]. Higher expressions of ST3GAL1 have also been found to be related to the advanced stage serous type epithelial ovarian cancer (EOC), and the process of a2,3-linked sialylation is highly important for clear cell type epithelial ovarian cancer (C-EOC) with the potential for a possible therapeutic solution [62].

In recent years, a large number of studies have suggested that upregulation of ST6GAL1 has been associated with tumor aggressiveness in various cancers. Such studies conducted for ovarian cancers using mRNA expression data suggest a strong correlation between high ST6GAL1 expression and lymphatic invasion of tumor cells, while western blot analysis of ST6GAL1 protein levels has suggested that patients diagnosed with distant metastasis often present significantly higher ST6GAL1 protein levels. The study further claims to be a promising therapeutic target, and metastasis formation might be decreased by blocking it due to its influence on hallmarks of the carcinogenic phenotype like adhesion, metastasis formation, and chemoresistance [63]. Studies have also described ST6GAL1′s complex relationship with Sox2, a master stem cell transcription factor. Located within the 3q26 amplicon, they are amplified in cancer cells, and due to the binding of the transcribed Sox2 to ST6GAL1, its expression is enhanced further. These events aid in the remodeling of cancer cells into a more stem-like cell phenotype, which itself is associated with ovarian cancer progression and recurrence [64]. Other sialyltransferases and their roles in the progression of ovarian cancer have also been explored. One such sialyltransferase is ST3GAL3, whose mRNA expression levels differed significantly amongst different ovarian cancer cell lines. HO8910PM with high invasive and metastatic capacity expressed elevated ST3Gal3 mRNA and displayed high chemoresistance to cisplatin, while in the case of SKOV3 cells, which have lower expression levels of ST3Gal3 show more chemo-sensitive to cisplatin. Their findings light on the reverse correlation between the expression levels of ST3GAL3 and the dosage of cisplatin used in various cell lines and claim that by targeting ST3GAL3, chemoresistance of cisplatin can be prevented the relapse of ovarian cancer [64]. Similar studies conducted have held ST3GAL3 accountable for paclitaxel-related resistance during ovarian cancer chemotherapy. They further suggest that overexpression of ST3Gal3 may serve as a diagnostic and prognostic marker and also a potential chemotherapeutic target for ovarian cancer [65]. Fewer studies also elaborate on the role of neuraminidases in ovarian cancer, one being where higher expression of NEU1 in ovarian cancer tissues of patients in comparison to adjacent normal tissues was noted. They further elucidate the role of NEU1 siRNA, which via targeting lysosome and oxidative phosphorylation signaling, effectively inhibits the proliferation, apoptosis, and invasion of human ovarian cancer cells suggesting NEU1 can be targeted for the treatment of ovarian cancer [66].

### 4.4. Other Cancers

Cytokines such as IL3 and CCL17, secreted by M2 macrophages co-cultured with colon cancer, induce expression of ST6GalNAc1, which results in higher expression of sTn antigen inducing on MUC1 [67]. ST6GalNAc6 downregulation in colon cancer is associated with epigenetic silencing, paralleled by decreased disialyl LeA expression with a concomitant increase in sialyl LeA [68]. Decreased expression of ST8Sia5 is associated with gene regulation by fork-head box O3 (FOXO3), the functional deficiency of which facilitates inflammation-mediated colon cancer growth linked to poor survival in patients [69]. In prostate cancer, high ST6Gal-I expression positively correlates with Gleason scores, seminal vesicle involvement, and poor survival in patients [70]. Shorter isoforms of ST6GalNAc1 (induced by androgens) exhibit a similar sialyltransferase activityClick or tap here to enter text., the former is responsible for expressing the sTn antigen [71]. ST6GalNAc3 is shown to be hypermethylated. However, it is not yet known if this epigenetic silencing contributes to the development of cancer [72]. In gastric cancer, ST3Gal4 contributes to poor prognosis and selectin-dependent adhesion through sLex, whereas ST6GalNAc 1 induces expression of sTn antigen and regulates the gene expression of IGF-1 through STAT5b activation [26,73]. ST6Ga1 expresses CDw75 antigen (a sialylated carbohydrate epitope), which strongly correlates with aggressive gastric tumors [74]. Other than this, ST6Gal1 and ST3Gal3 expression has also shown an association with secondary local tumor recurrences in gastric cancer (*p* = 0.005; *p* = 0.012) [75]. In renal cancer, silencing ST6GalNAc6 increases the metastatic capability of tumor cells, expressing low levels of DSGb5, and decreasing migration, but not proliferation [76].

Another cancer with an increasing incidence and extremely high mortality rates is the one primarily affecting Esophagus [77]. Significant downregulation of ST6GALNAC1 in Esophageal Squamous Cell Carcinoma (ESCC) tissues in comparison to normal tissues via hyper-methylation and loss of heterozygosity have been reported, possibly making ST6GALNAC1 a candidate responsible gene for ESCC [78]. Another study conducted with Cancer Stem Cells (CSCs), the primary cause of cancer recurrence and metastasis, found ST8SIA2 to be hypermethylated in ESCC-CSCs. Further analysis conducted with GEPIA also revealed ST8SIA2 to be highly expressed in ESCC tissues in comparison to normal tissues and also correlated with poor disease-free survival [79]. Another study involving Adenoviral- fragile histidine triad (Ad-FHIT) treatment of esophageal cancer cells found overexpression of ST3GAL6 in apoptotic cells compared with non-apoptotic cells, suggesting Ad-FHIT treatment’s role in the inhibition of a sialyltransferase-associated metastasis [80]. mRNA expression analysis of various sialyltransferases and neuraminidases was conducted using RT-qPCR in Oral Squamous Cell Carcinoma (OSCC), found sialyltransferases ST3GAL1, ST3GAL2, ST3GAL3, ST3GAL4, ST3GAL6, and ST6GAL1 and neuraminidase NEU3 to be downregulated in tumor tissues in comparison to normal. Further analysis revealed the correlation between elevated levels of ST3GAL2 and ST3GAL3 with metastasis and progression of OSCC [81].

In the case of Head and Neck Squamous Cell Carcinoma (HNSCC), elevated mRNA levels of NEU3 were found in tumor tissues in comparison to normal tissues. The elevation was also found to be associated with lymph node metastasis, which is an important event for HNSCC progression and is often related to higher mortality rates. Further studies to understand the underlying mechanism reveal that NEU3 participates in HNSCC progression through EGFR signaling regulation and can be targeted to inhibit the disease progression [82]. Studies have revealed that cell adhesion and invasion in Anaplastic Large Cell Lymphoma (ALCL) are regulated by the process of sialylation involving ST6Gal1 and N-glycans. Galectin-8 causes the growth inhibition of lymphoma cells induced by neuraminidase treatment [83]. Another study’s findings suggest that in the case of lymphoreticular malignancies, an increase in sialyltransferase activity is observed [84].

Sialyltransferase studies revolving around various types of skin cancers have also surfaced, especially around melanoma, the most serious form of skin cancer. Studies report that ST3GAL1 promotes melanoma metastasis via AXL and also acts as a target for the oncogenic SOX2-GLI1 transcriptional complex [85]. A study involving the evaluation of sialyltransferase expression in actinic keratosis, keratoacanthoma, squamous cell carcinoma, and basal cell carcinoma found elevated levels of ST3Gal1 and ST6Gal1 to be associated with skin tumors with greater potential for invasion and metastasis [86]. 

The most frequent malignant tumor of the urinary system is Bladder Cancer (BLCA), and the leading cause of death from the disease is distant metastasis. Data from the Gene Expression Profiling Interactive Analysis and TCGA databases revealed that ST8SIA1′s mRNA expression levels were lower in BLCA tissues than in healthy tissues, and this was further supported by immunohistochemistry and western blot analysis. The pathogenic grade and invasiveness of BLCA were adversely correlated with the ST8SIA1 expression levels. Western blot analysis showed that BLCA cell lines expressed ST8SIA1 at lower levels than a typical urothelial cell line. The proliferation, migration, and invasion of T24 and 5637 BLCA cells were inhibited by ST8SIA1 overexpression, according to CCK-8, flow cytometry, wound healing, colony formation, and Transwell experiments. Further research showed that ST8SIA1 might reduce the production of JAK/STAT pathway-targeting signal molecules such as MMP2, proliferating cell nuclear antigen, cyclin D1, and Bcl2 in two BLCA cell lines, as well as suppress the phosphorylation of JAK2 and STAT3. This gave researchers a fresh target for the detection and therapy of BLCA [87]. 

One of the most common tumors that kill female patients is Cervical Cancer, which also has the second-highest mortality rate among cancers in underdeveloped nations [88]. Cervical cancer has been shown to be brought on by Human Papillomavirus (HPV) infection [89]. Recent research showed that ST3Gal IV levels were lower in cervical cancer tissues than in nearby tissues and were inversely linked with the tumor’s aggressiveness. In vitro and in vivo, ST3Gal IV overexpression reduced the growth and division of cervical cancer HeLa and SiHa cells. Additionally, ST3Gal IV overexpression increased the expression of various Notch pathway constituents, including Jagged1, Notch1, Hes1, and Hey1, while decreasing the expression of cell cycle proteins, including Cyclin D1, Cyclin E1, CDK2, and CDK4. These findings suggest that the Notch/p21/CDKs signaling pathway negatively regulates cell proliferation in cervical cancer by reducing ST3Gal IV expression. As a result, ST3GalIV may be a target for the detection and treatment of cervical cancer [90].

A hematopoietic stem cell condition called Chronic Myeloid Leukemia (CML) results from a translocation of chromosomes 9 and 22′s long arms, which contains the BCR-ABL fusion oncogene. Recent studies have shown that ST8SIA4 mRNA had the highest level of expression and was a major factor in the high levels of 2,8-linked NeuNAc residues on the PBMC surfaces of CML/MDR patients, but ST8SIA6 expression was at a high level in CML patients, indicating ST8SIA6 to be an essential factor in the emergence of malignant changes. In CML cell lines, ST8SIA4 altered expression significantly reduced PI3K/Akt pathway activity [91]. Additional research showed that altered ST3GALIV expression had a substantial impact on the distribution of the cell cycle, the apoptotic signal, and cell proliferation [92].

According to a recent study, ST6GAL1 levels dramatically rose, whereas ST3GAL1, ST6GALNAC3, and ST8SIA6 levels were noticeably decreased in lung cancer tissues and cells. Lung cancer tissues and cells have increased ST6Gal-I mRNA, protein, and glycan levels. Additionally, down-regulating ST6Gal-I reduced the levels of the proteins Jagged1, DLL-1, Notch1, Hes1, Hey1, Matrix-Metalloproteinases (MMPs), and VEGF. It also inhibited the ability of A549 and H1299 cells to proliferate, migrate, and invade in vitro. Additionally, ST6Gal-I silencing of A549 and H1299 cells resulted in reduced proliferation and metastasis, which was reversed by Notch1 overexpression. This suggests that ST6Gal-I may mediate the invasiveness and tumorigenicity of NSCLC cells via the Notch1/Hes1/MMPs pathway both in vitro and in vivo. Modification of 2,6-sialylation is positively associated with lung cancer development [93].

## 5. Resistance against Traditional Cancer Treatments

Chemotherapeutics and radiotherapy are among the top traditional methods for treating cancer. It has been observed by the work of several groups that sialyltransferases and neuraminidases tend to create resistance in the treatment of various cancers.

### 5.1. Chemoresistance

The capacity of cancer cells to tolerate the presence of therapies is a crucial component of their malignant nature. It has been demonstrated that hypersialylation of cancer cells encourages chemoresistance in a number of cancer types. This characteristic has received particular attention in regard to ST6GAL1, which can directly sialylate and activate EGFR, FGFR1, and HER2. The EGFR inhibitor gefitinib had a greater anti-cancer effect on colon cancer cells when ST6GAL1 was silenced, but ST6GAL1 overexpression reduced gefitinib’s cytotoxic effects [94]. Schultz and colleagues demonstrated that ST6Gal I is highly expressed in cisplatin-resistant cells compared with nonresistant cells [95]. Other studies have also reported that overexpression of ST6Gal1 confers resistance to cisplatin, a platinum-based chemotherapeutic drug. They also concluded that ST6Gal1 is responsible for the regulation of cancer stem cell resistance to irinotecan, another chemotherapeutic drug [52]. It is also observed to mediate resistance in chemoradiotherapy in rectal cancer due to the inhibition of apoptosis [96]. Chemoresistance is also promoted by the ST3GAL family. Overexpression of ST3GAL1 improved paclitaxel resistance in ovarian cancer cells, but ST3GAL1 downregulation had the reverse effect [23]. ST3GAL1 was found to Mediate Drug Resistance (MDR) in Chronic Myeloid Leukemia (CML) cells. Compared to CML with MDR, ST3GAL1 is markedly increased in CML without MDR. While ST3GAL1 overexpression reduces chemoresistance in KCL22/ADR cells, ST3GAL1 silencing causes MDR in KCL22 cells. Additionally, gastric adenocarcinoma MKN45 spheroids are more resistant to Crizotinib, a medication that inhibits the receptor tyrosine kinases RON and MET, due to overexpression of ST3GAL4 and ST3GAL6 [97]. Through ST3GAL5 and ST8SIA4, which are both highly expressed in drug-sensitive cells and Adriamycin-resistant cells, sialylation plays a role in the establishment of MDR of AML cells. In HL60 cells, ST3GAL5 suppression or ST8SIA4 overexpression increases the expression of P-glycoprotein (P-gp) and MDR-related protein 1 (MRP1), which promotes the development of MDR in AML cells [98]. Additionally, it has been demonstrated that ST8SIA1 is increased in patients with chemo-resistant TNBC, and its inhibition makes TNBCs more susceptible to chemotherapy by inhibiting the Wnt/-catenin and FAK/Akt/mTOR pathways [99].

In the ST6GALNAC family, it was discovered that overexpressing ST6GALNAC1 caused MKN45 gastric cancer cells to express sTn and conferred resistance to cisplatin or 5-fluorouracil (5-FU), whereas its knockdown restored galectin-3-binding sites and made tumor cells more susceptible to drug-induced cell death [100]. Additionally, ST6GALNAC2 silencing reduced the 5-fluorouracil (5-FU)-induced chemoresistance of HCT-8 and LoVo colorectal cancer cells to miR-135b or miR-182 suppression [101]. ST6GalNAc2 has been associated with the PI3K/AKT pathway to mediate the invasiveness as well as the resistance of colorectal cancer to 5-fluorouracil (5-FU) [102].

ST3Gal2 has been observed to be a rate-limiting enzyme for SSEA-4 (sialyl-glycolipid stage-specific embryonic antigen 4) synthesis, which has shown a positive correlation with chemoresistance [103,104]. ST3Gal3 has shown resistance to paclitaxel and cisplatin resistance in ovarian cancer cells [65,105]. NEU1 has been shown to regulate MUC1 activity, which is responsible for inducing drug resistance in human (BxPC3 and Capan-1) and mouse (KCKO, KCM) pancreatic cancer cells [106,107].

### 5.2. Radiotherapy 

Exposure to radiotherapy has shown induction of high ST6Gal1 expression (in a dose-dependent manner) both among healthy and cancerous tissues. This radiation could be further linked with increased α2-6 sialylation of β1-integrins, leading to increased cell migration and adhesion. In another study, ST6Gal1 induced radio-resistance, and when Si-ST6Gal-I or Neu2 was co-transfected, the increased radio-resistance was abolished, suggesting that ST6Gal-I mediated protein sialylation is involved in the radiation resistance response and protein sialylation enables the cell to resist radiation-induced damage through the inhibition of apoptosis [108]. Other than this, irradiation also induced the expression of other sialyltransferases (ST3Gal I-IV, ST8Sia I) [108,109].

## 6. Sialyltransferase Inhibitors

Sialyltransferases and their sialylation processes serve as potentially important targets in strategies designed to identify and develop treatments for cancer metastasis, given the substantial body of biological evidence that they are connected with tumor progression, adhesion, and migration in human cancer cells [2].

The initial tumor is not the main cause of cancer-related patient mortality; metastasis is the main cause (>90%). The cancer spreads from the primary tumor location through Extracellular Matrix (ECM) and basement membrane barriers before reaching other organs through metastatic processes, which work through lymph nodes and blood transmission channels. Multiple biological processes, including tumor cell attachment, degeneration, and migration, are involved in the spread of cancer. A therapeutic approach for the management of cancer metastasis could be the inhibition of any one of these phases, which could stop the complete metastatic cycle [110]. A promising class of compounds that may be utilized to treat cancer metastasis clinically includes powerful, cell-permeable, and subtype-selective sialyltransferase inhibitors. These agents function by blocking sialyltransferase-mediated hypersialylation of cell surface glycoproteins or glycolipids, which subsequently obstructs the sialic acid recognition pathway and impairs cell motility and invasion [2]. 

The drug imatinib belongs to the group of drugs known as kinase inhibitors. It functions by preventing the aberrant protein from signaling the growth of cancer cells. This slows the growth of cancerous cells. Imatinib-acquired resistance is usually linked to poor clinical outcomes for patients with Chronic Myeloid Leukemia (CML). Recent research has shown that K562R cells (CML cells with imatinib resistance phenotype) have higher sialylation levels than K562 cells. It was found that imatinib-resistant K562R cells had much higher levels of CMP-N-acetylneuraminate-beta-galactosamide-alpha-2,3-sialyltransferase (ST3Gal IV) than imatinib-susceptible K562 cells using qRT-PCR and western blotting research [92]. 

Using 4-methylumbellipheryl-labeled LacNAc as the acceptor substrate, Ogawa and colleagues discovered the methyl 5a’-carbadisaccharides, in which the monosaccharide units are connected via ether or amine bridges, possessed significant inhibitory activities (IC50 = 185-419 M) against recombinant 2,3-sialyltransferases [111]. Cytidine diphosphate (CDP) mimics the CMP (Cytidine 5′-Monophosphate) part of the donor substrate CMP-Neu5Ac and is a strong competitive inhibitor of sialyltransferases (Ki = 10 M). Despite their effectiveness, CDP and its equivalents have not been thoroughly investigated biologically as ST inhibitors [112]. As polysialyltransferase inhibitors, researchers created the donor analogs 5-methyl CMP and 2′-O-methyl CMP. Their findings showed that ST8Sia-IV, ST8-Sia-II, and ST8Sia-III were all severely inhibited by 2′-O-methyl CMP [113,114]. The inhibitors inhibited the expression of polysialic acid on the cell surface. Bisubstrate-type compounds, such as those that include both donor (CMP-Neu5Ac) and acceptor (N-acetyllactosamine, LacNAc) components, have previously been recognized for their use as sialyltransferase inhibitors [115]. Currently, efforts are concentrated on using high-throughput screening (HTS) techniques to quickly identify powerful protein inhibitors, particularly ST inhibitors. The MS-based, quick, and quantitative screening method created by Nishimura and colleagues is particularly appealing in this regard. With this technique, the inhibitory effects of drugs on STs were quantified while CMP-Neu5Ac and a standard internal acceptor that was labeled with a stable isotope were present (OCD3) [116]. 

Tsai and colleagues found that soyasaponin-I, a naturally occurring substance made from raw soybean saponin, served as a highly effective ST3Gal-I inhibitor [117]. Additionally, soyasaponin-I modified the invasive behavior of tumor cells by specifically suppressing the mRNA expression of ST3Gal-IV and reducing the expression of 2,3-sialic acid on tumor cell surfaces. Soyasaponin I efficiently and selectively reduced 2,3-sialylation on the cell surface, decreased cancer cell migration, and improved cell adherence to extracellular matrix proteins in the highly metastatic cancer cell line B16F10 [118,119]. Administration of soyasaponin-I was discovered to prevent lung metastasis in mice, indicating that the natural product changed the sialylation of cell surface adhesion molecules, preventing tumor cells from spreading to the lungs significantly. It has also been acknowledged that plant-derived flavonoids are useful as anti-tumor, anti-inflammatory, antibacterial, and antioxidant agents [120,121,122,123,124]. Members of this inhibitor family have tetrahydropyran or dihydropyranone C-ring structures with two phenyl groups (A-ring and B-ring) linked by three carbon atoms and one oxygen atom. Due to the wide range of biological actions exhibited by them, flavonoids may also have an impact on processes that are connected to cancer. Several flavonoid derivatives with three different types of core structures were described by Suzuki and coworkers. Their findings revealed that several of these flavonoid analogs significantly inhibited the growth of rat ST6Gal-I, human ST6Gal-I, and rat ST3Gal-III [125]. Further information regarding sialyltransferase inhibitors can be found in Table 3.

## 7. Neuraminidase Inhibitors

Neu5Ac (5-acetamido-3,5-dideoxy-D-glycero-D-galacto-non-2-ulosonic acid) is the most prevalent type of neuraminic acid present in the human body [156]. Neuraminic acids, also known as sialic acid, often serve as the last residues in glycan chains and are essential for molecular recognition [157]. Strong and specific chemical inhibitors act as useful tools for examining how these enzymes function biologically. The development of a powerful neuraminidase inhibitor has received considerable attention; however, research into inhibitors of human neuraminidases has lagged behind. According to reports, 2-deoxy-2,3-didehydro-N-acetylneuraminic acid (DANA) is the basis of the majority of human neuraminidase inhibitors [158]. It is interesting to notice that when Neu5Ac is present in sufficient quantities, the NEU enzymes can produce DANA. The dehydration caused by Neu5Ac when it is bound in the active site is probably what causes DANA to develop [159].

According to a recent study, it was discovered how the scaffold of the neuraminidase inhibitor, DANA, can be exploited as a starting point for the creation of novel powerful, and selective inhibitors of human neuraminidase isoenzymes. The study focuses on how the presence of an aryltriazolyl group at the C9 position greatly improved the potency of inhibitors towards NEU3 and NEU4. Further, it was also discovered how the addition of a guanidino group at the C4 position provided the selectivity between NEU3 and NEU4, revealing a clear strategy for the design of inhibitors with improved selectivity for NEU3 and sub-micromolar potencies. These inhibitors could be made into practical instruments for researching how human neuraminidases work biologically. In conclusion, it was proven that distinct structural characteristics of the human neuraminidase isoenzymes’ active site enabled the discovery of highly effective and selective inhibitors of this significant family of human glycosidases. Future research will focus on how these substances behave in living organisms and develop new instruments for analyzing the precise biological functions of NEU isoenzymes [155]. Though most of the research on neuraminidase inhibitors focuses on influenza viruses and other infectious diseases, a 2016 research study on a novel GPCR-MMP9-NEU1 signaling pathway found it to be probably essential for tumor development. They further demonstrated that the therapeutic effectiveness of oseltamivir phosphate targeting NEU1 could restrict the tumor’s capacity to metastasize [160]. Another study regarding anti-influenza sialidase inhibitor oseltamivir phosphate found it to reduce the neuraminidase activity of canine mammary cancer cells and contribute to an increase in mammary tumor aggressiveness [152]. An improvement in the outcome of Leukemia patients has also been observed with neuraminidase inhibitor studies involving oseltamivir and zanamivir [153]. 

Furthermore, not only are neuraminidase inhibitors being used for the treatment of cancer but engineered neuraminidases have been identified to improve disialylation and serve as a cancer immunotherapeutic approach. One such example of that of engineered PD-L1-targeted sialidases which have been characterized in preclinical models and found to have the capability to improve disialylation of PD-L1-expressing tumor and immune cells [150]. Another such case is that of E-602, a novel cancer drug candidate with the potential to act as a sialoglycan degrader and is currently under Phase 1/2 studies of its clinical trials. It is an engineered human sialidase (Neu2) Fc fusion that restores the function of T cells by desialylating surface sialoglycans on tumor and immune cells [151,161]. 

Precision glycocalyx editing by the development of antibody–sialidase conjugates have also been proposed to be a potential cancer immune therapy. A study involving the fusion of recombinant neuraminidase to HER2-specific antibody trastuzumab was found to selectively desialylate of the tumor cell glycocalyx and increase the cell susceptibility to Antibody-Dependent Cell-mediated Cytotoxicity (ADCC) and hence serve as a treatment for those cancer patients having lower levels of HER2 or resistance to trastuzumab [149]. Biotechnology companies such as Palleon Pharmaceuticals and Shanghai Henlius Biotech also show an increasing interest in using sialidases for potential cancer treatment. This can be estimated by their press release which indicates their plans to develop a Bifunctional HER2-Sialidase and a second bifunctional sialidase for the degradation of Immunosuppressive Sialoglycans for Cancer. The earlier mentioned clinical trials of novel drugs E-602 are also being conducted and sponsored by Palleon Pharmaceuticals [154,161]. 

## 8. Conclusions

This review focuses on the significance of Sialyltransferase and Neuraminidase in cancer prognosis and has highlighted the various Sialyltransferase and Neuraminidase families, such as ST3GAL, ST6GAL, ST6GALNAC, ST8SIA, NEU, involved in the metastasis of various cancers such as breast, pancreatic, ovarian, head and neck, etc. The compilation of experimental and computational data presented in this paper provides researchers working on particular cancers with possible ST/NEU targets or biomarkers for further studies. This review also shed insight on the range of novel Sialyltransferase inhibitors now under development, emphasizing recent iterative accomplishments in designing and developing new inhibitors using the CMP-Neu5Ac frameworks. The necessity for potent and selective molecules is highlighted by the aberrant sialylation process involving STs and Neu compounds contributing to the development and spread of cancer. Obtaining protein for the required in vitro experiments, as well as determining whether the inclusion of a negative charge and numerous polar groups in CMP-sialic acid mimics will be favorable to cell uptake and oral bioavailability, are among the problems facing ST inhibition. Further research in related areas will allow researchers to identify the most efficient target amongst the presented sialyltransferases and neuraminidases in different cancers and gain structural, mechanistic, and computational knowledge, which will further aid in designing potent compounds targeting these enzymes. 

## Figures and Tables

**Table 1:** Human sialyltransferases (STs). Other names used to refer to them, the substrate carrier, along with the structure formed as a result of the ST activity, have been mentioned in the table below.

Enzymes	Other Names	Substrate Carrier	Structure Formed
ST3Gal1	SIAT4A, ST3O, ST3GalA.1	O-GP > GL	Neu5Acα2-3Galβ1-3GalNAc-
ST3Gal2	SIAT4B, ST3GalA.2, SAT4	GL > O-GP	Neu5Acα2-3Galβ1-3GalNAc-
ST3Gal3	SIAT6	GP	Neu5Acα2-3Galβ1-3/4GlcNAcβ-
ST3Gal4	SIAT4, STZ, SAT3, SIAT4C	GP > GL	Neu5Acα2-3Galβ1-3GalNAc- Neu5Acα2-3Galβ1-4GlcNAc-
ST3Gal5	SIAT9, SIATGM3S	GL	Neu5Acα2-3Galβ1-4Glc-Cer
ST3Gal6	SIAT10	GP, GL	Neu5Acα2-3Galβ1-4GlcNAcβ-
ST6Gal1	SIAT1	N-GP, OL, GL	Neu5Acα2-6Galβ1-4GlcNAcβ-
ST6Gal2	SIAT2	N-GP, OL	-
ST6GalNAc1	SIAT7A	O-GP	(Neu5Acα2-3)_0-1_(Galβ1-3)_0-1_GalNAc-Ser/Neu5Acα2-6
ST6GalNAc2	SIAT7, SIAT7B, SIATL1	O-GP	(Neu5Acα2-3)_0-1_Galβ1-3GalNAc-Ser/Neu5Acα2-6
ST6GalNAc3	SIAT7C	O-GP, GL	Neu5Acα2-3Galβ1-3GalNAc-R/Neu5Acα2-6
ST6GalNAc4	SIAT3C, SIAT7D	O-GP, OL > GL	Neu5Acα2-3Galβ1-3GalNAc-R/Neu5Acα2-6
ST6GalNAc5	SIAT7E	GL	G_D1α_
ST6GalNAc6	SIAT7F	GL > GP	G_D1α_, (G_T1α_)
ST8Sia1	SIAT8, SIAT8A	GL	Neu5Acα2-8Neu5Acα2-3Galβ1-4Glc-Cer
ST8Sia2	SIAT8B, ST8SIA-II, STX	GP	Neu5Acα2-8Neu5Acα2-3Galβ1-4GlcNAc
ST8Sia3	SIAT8C	GP, GL	Neu5Acα2-8Neu5Acα2-3Gal1-4GlcNAc
ST8Sia4	SIAT8D	GP	Neu5Acα2-8(Neu5Acα2-8) _nNeu_5Acα2-3Galβ1-R
ST85Sia5	SIAT8E	GL	GD1c, GT1a, GQ1b, GT3
ST8Sia6	SIAT8F	O-GP, OL	-

GL = glycolipid, GP = glycoprotein, OL = oligosaccharides, O-GP = O-glycosylated protein, N-GP = N-glycosylated protein.

**Table 2:** Human Neuraminidases (NEUs). Information regarding alternative names, substrate carrier, intracellular localization, etc., corresponding to NEUs can be found in the table below.

Enzymes	Other Names	Substrate Carrier	Tissue Expression	Intracellular Localization	Glycosidic Linkage Specificity
NEU1	Lysosomal sialidase, SIAL1	OL, glycopeptides	Kidney, pancreas, skeletal muscle, liver, lungs, placenta, and brain	Lysosomal and plasma membranes	α2-3 faster than α2-6 and α2-8 [10]
NEU2	Cytosolic sialidase, SIAL2	OL, GP, GL	Skin	Cytosol	α2-3 faster than α2-6 and α2-8 [10]
NEU3	Membrane sialidase, SIAL3	GL, Grb2	Adrenal gland, skeletal muscle, heart, testis, and thymus	Caveolae microdomains of plasma, endosomal and lysosomal membranes	α2-3 and α2-8 almost equally and faster than α2-6 [11]
NEU4	Sialidase	OL, GP, GL, phospholipid scramblase 1	Brain, skeletal muscle, heart, placenta, and liver	ER membrane, mitochondria, and lysosomes	-

GP = glycoproteins, GL = gangliosides, Grb2 = growth factor receptor bound protein 2.

**Table 3:** Important enzymes involved in sialylation, their role in different cancers, and respective treatment strategies.

Enzyme	Type of Cancer in Which the Enzyme is Involved	Treatment/Drug
ST3GAL1	Breast Cancer [37], Leukemia [126], Bladder cancer [127], RCC [128], Melanoma [85], Ovarian cancer [23]	Hexapeptide NH2GNWWWW [129], Stachybotrydial [130], Lith-O-Asp (Pan ST) [131], Soyasaponin-I [117,118]
ST3GAL3	Breast Cancer [24]	Lith-O-Asp analogues FCW34 and FCW66 [132]
ST3GAL4	Pancreatic cancer [60], Cervical cancer [133], Leukemia [92]	Soyasaponin-I [119]
ST3GAL6	Bladder cancer [134]	3Fax-Neu5Ac [135]
ST6GAL1	Colon cancer [136], Rectal cancer [96], Prostate cancer [70], Breast cancer [38], Gastric cancer [137], Pancreatic cancer [138], Cervical cancer [139], RCC [140], Ovarian cancer [63]	-
ST6GALNAC1	Gastric cancer [141], ESCC [78]	-
ST6GALNAC2	Breast [142]	-
ST8SIA Family	Breast cancer [42], Bladder cancer [87]	2′-O-methyl CMP [114]
NEU Family	Gastric cancer [143], Pancreatic cancer [59,144] Bladder cancer [145], Ovarian cancer [66], HNSCC [82], RCC [146], Lung cancer [147,148]	Sialidase conjugation to trastuzumab (Precision glycocalyx editing) [149], Engineered PD-L1-targeted sialidase [150], Bi-Sialidase E602-GLIMMER-01 (Glycan-Mediated Immune Regulation) study [151], Oseltamivir [152], Zanamivir [153], Bifunctional HER2-Sialidase [154], DANA Analogues [155]

## Data Availability

Not applicable.

## References

[1] Weir H.K., Thompson T.D., Stewart S.L., White M.C. (2021). Cancer Incidence Projections in the United States between 2015 and 2050. Prev. Chronic Dis..

[2] Perez S.J.L.P., Fu C.-W., Li W.-S. (2021). Sialyltransferase Inhibitors for the Treatment of Cancer Metastasis: Current Challenges and Future Perspectives. Molecules.

[3] Dobie C., Skropeta D. (2021). Insights into the role of sialylation in cancer progression and metastasis. Br. J. Cancer.

[4] Reily C., Stewart T.J., Renfrow M.B., Novak J. (2019). Glycosylation in health and disease. Nat. Rev. Nephrol..

[5] Li F., Ding J. (2019). Sialylation is involved in cell fate decision during development, reprogramming and cancer progression. Protein Cell.

[6] Pietrobono S., Stecca B. (2021). Aberrant Sialylation in Cancer: Biomarker and Potential Target for Therapeutic Intervention?. Cancers.

[7] Burger P.C., Lötscher M., Streiff M., Kleene R., Kaissling B., Berger E.G. (1998). Immunocytochemical Localization of α2,3(N)-sialyltransferase (ST3Gal III) in Cell Lines and Rat Kidney Tissue Sections: Evidence for Golgi and Post-Golgi Localization. https://academic.oup.com/glycob/article-abstract/8/3/245/560307.

[8] Li Y., Chen X. (2012). Sialic acid metabolism and sialyltransferases: Natural functions and applications. Appl. Microbiol. Biotechnol..

[9] Louten J. (2016). Influenza Viruses. Essential Human Virology.

[10] Miyagi T., Tsuiki S. (1984). Rat-liver lysosomal sialidase. Solubilization, substrate specificity and comparison with the cytosolic sialidase. JBIC J. Biol. Inorg. Chem..

[11] Miyagi T., Tsuiki S. (1985). Purification and Characterization of Cytosolic Sialidase from Rat Liver. J. Biol. Chem..

[12] Büll C., Stoel M.A., Brok M.H.D., Adema G.J. (2014). Sialic Acids Sweeten a Tumor’s Life. Cancer Res..

[13] Picco G., Julien S., Brockhausen I., Beatson R., Antonopoulos A., Haslam S., Mandel U., Dell A., Pinder S., Taylor-Papadimitriou J. (2010). Over-expression of ST3Gal-I promotes mammary tumorigenesis. Glycobiology.

[14] Cheng J., Wang R., Zhong G., Chen X., Cheng Y., Li W., Yang Y. (2020). ST6GAL2 Downregulation Inhibits Cell Adhesion and Invasion and is Associated with Improved Patient Survival in Breast Cancer. OncoTargets Ther..

[15] Yu X., Wu Q., Wang L., Zhao Y., Zhang Q., Meng Q., Wang S. (2016). Silencing of ST6GalNAc I suppresses the proliferation, migration and invasion of hepatocarcinoma cells through PI3K/AKT/NF-κB pathway. Tumor Biol..

[16] Ma X., Dong W., Su Z., Zhao L., Miao Y., Li N., Zhou H., Jia L. (2016). Functional roles of sialylation in breast cancer progression through miR-26a/26b targeting ST8SIA4. Cell Death Dis..

[17] Kroes R.A., He H., Emmett M.R., Nilsson C.L., Leach F.E., Amster I.J., Marshall A.G., Moskal J.R. (2010). Overexpression of ST6GalNAcV, a ganglioside-specific α2,6-sialyltransferase, inhibits glioma growth in vivo. Proc. Natl. Acad. Sci. USA.

[18] Schnaar R.L., Gerardy-Schahn R., Hildebrandt H. (2014). Sialic acids in the brain: Gangliosides and polysialic acid in nervous system development, stability, disease, and regeneration. Physiol. Rev..

[19] Welch D.R., Hurst D.R. (2019). Defining the Hallmarks of Metastasis. Cancer Res..

[20] Dongre A., Weinberg R.A. (2019). New insights into the mechanisms of epithelial-mesenchymal transition and implications for cancer. Nat. Rev. Mol. Cell Biol..

[21] Wu H., Shi X.-L., Zhang H.-J., Hu W.-D., Mei G.-L., Chen X., Song Q.-J., Mao Q.-S., Chen Z., Yang X.-B. (2016). Overexpression of ST3Gal-I promotes migration and invasion of HCCLM3 in vitro and poor prognosis in human hepatocellular carcinoma. OncoTargets Ther..

[22] Wen K.-C., Sung P.-L., Hsieh S.-L., Chou Y.-T., Lee O.K.-S., Wu C.-W., Wang P.-H. (2017). α2,3-sialyltransferase type I regulates migration and peritoneal dissemination of ovarian cancer cells. Oncotarget.

[23] Wu X., Zhao J., Ruan Y., Sun L., Xu C., Jiang H. (2018). Sialyltransferase ST3GAL1 promotes cell migration, invasion, and TGF-β1-induced EMT and confers paclitaxel resistance in ovarian cancer. Cell Death Dis..

[24] Cui H.-X., Wang H., Wang Y., Song J., Tian H., Xia C., Shen Y. (2016). ST3Gal III modulates breast cancer cell adhesion and invasion by altering the expression of invasion-related molecules. Oncol. Rep..

[25] Pérez-Garay M., Arteta B., Pagès L., de Llorens R., de Bolòs C., Vidal-Vanaclocha F., Peracaula R. (2010). α2,3-Sialyltransferase ST3Gal III Modulates Pancreatic Cancer Cell Motility and Adhesion In Vitro and Enhances Its Metastatic Potential In Vivo. PLoS ONE.

[26] Guerrero P.E., Miró L., Wong B.S., Massaguer A., Martínez-Bosch N., De Llorens R., Navarro P., Konstantopoulos K., Llop E., Peracaula R. (2020). Knockdown of α2,3-Sialyltransferases Impairs Pancreatic Cancer Cell Migration, Invasion and E-selectin-Dependent Adhesion. Int. J. Mol. Sci..

[27] Rodríguez E., Schetters S.T.T., Van Kooyk Y. (2018). The tumour glyco-code as a novel immune checkpoint for immunotherapy. Nat. Rev. Immunol..

[28] Büll C., Boltje T.J., Balneger N., Weischer S.M., Wassink M., van Gemst J.J., Bloemendal V.R., Boon L., van der Vlag J., Heise T. (2018). Sialic Acid Blockade Suppresses Tumor Growth by Enhancing T-cell–Mediated Tumor Immunity. Cancer Res..

[29] Perdicchio M., Cornelissen L.A.M., Streng-Ouwehand I., Engels S., Verstege M.I., Boon L., Geerts D., van Kooyk Y., Unger W.W.J. (2016). Tumor sialylation impedes T cell mediated anti-tumor responses while promoting tumor associated-regulatory T cells. Oncotarget.

[30] Wang L., Li S., Yu X., Han Y., Wu Y., Wang S., Chen X., Zhang J., Wang S. (2019). α2,6-Sialylation promotes immune escape in hepatocarcinoma cells by regulating T cell functions and CD147/MMP signaling. J. Physiol. Biochem..

[31] Locksley R.M., Killeen N., Lenardo M.J. (2001). The TNF and TNF Receptor Review Superfamilies: Integrating Mammalian Biology. Cell.

[32] Swindall A.F., Bellis S.L. (2011). Sialylation of the Fas Death Receptor by ST6Gal-I Provides Protection against Fas-mediated Apoptosis in Colon Carcinoma Cells. J. Biol. Chem..

[33] Holdbrooks A.T., Britain C.M., Bellis S.L. (2018). ST6Gal-I sialyltransferase promotes tumor necrosis factor (TNF)-mediated cancer cell survival via sialylation of the TNF receptor 1 (TNFR1) death receptor. J. Biol. Chem..

[34] Jiang Y., Wen T., Yan R., Kim S., Stowell S.R., Wang W., Wang Y., An G., Cummings R.D., Ju T. (2020). O-glycans on death receptors in cells modulate their sensitivity to TRAIL-induced apoptosis through affecting on their stability and oligomerization. FASEB J..

[35] Siegel R.L., Miller K.D., Jemal A. (2018). Cancer statistics, 2018. CA Cancer J. Clin..

[36] Recchi M.A., Hebbar M., Hornez L., Harduin-Lepers A., Peyrat J.P., Delannoy P. (1998). Multiplex reverse transcription polymerase chain reaction assessment of sialyltransferase expression in human breast cancer. Cancer Res..

[37] Burchell J., Poulsom R., Hanby A., Whitehouse C., Cooper L., Clausen H., Miles D., Taylor-Papadimitriou J. (1999). An 2,3 sialyltransferase (ST3Gal I) is elevated in primary breast carcinomas. Glycobiology.

[38] Hait N.C., Maiti A., Wu R., Andersen V.L., Hsu C.-C., Wu Y., Chapla D.G., Takabe K., Rusiniak M.E., Bshara W. (2022). Extracellular sialyltransferase st6gal1 in breast tumor cell growth and invasiveness. Cancer Gene Ther..

[39] Sewell R., Bäckström M., Dalziel M., Gschmeissner S., Karlsson H., Noll T., Gätgens J., Clausen H., Hansson G.C., Burchell J. (2006). The ST6GalNAc-I Sialyltransferase Localizes throughout the Golgi and Is Responsible for the Synthesis of the Tumor-associated Sialyl-Tn O-Glycan in Human Breast Cancer. J. Biol. Chem..

[40] Murugaesu N., Iravani M., van Weverwijk A., Ivetic A., Johnson D.A., Antonopoulos A., Fearns A., Jamal-Hanjani M., Sims D., Fenwick K. (2014). An In Vivo Functional Screen Identifies ST6GalNAc2 Sialyltransferase as a Breast Cancer Metastasis Suppressor. Cancer Discov..

[41] Drolez A., Vandenhaute E., Delannoy C.P., Dewald J.H., Gosselet F., Cecchelli R., Julien S., Dehouck M.-P., Delannoy P., Mysiorek C. (2016). ST6GALNAC5 Expression Decreases the Interactions between Breast Cancer Cells and the Human Blood-Brain Barrier. Int. J. Mol. Sci..

[42] Kan J.-Y., Moi S.-H., Hung W.-C., Hou M.-F., Chen F.-M., Shih S.-L., Shiau J.-P., Li C.-L., Chiang C.-P. (2020). Comprehensive Transcriptomic Analysis Identifies *ST8SIA1* as a Survival-Related Sialyltransferase Gene in Breast Cancer. Genes.

[43] Ruckhäberle E., Karn T., Rody A., Hanker L., Gätje R., Metzler D., Holtrich U., Kaufmann M. (2009). Gene expression of ceramide kinase, galactosyl ceramide synthase and ganglioside GD3 synthase is associated with prognosis in breast cancer. J. Cancer Res. Clin. Oncol..

[44] Battula V.L., Shi Y., Evans K.W., Wang R.-Y., Spaeth E., Jacamo R.O., Guerra R., Sahin A.A., Marini F.C., Hortobagyi G. (2012). Ganglioside GD2 identifies breast cancer stem cells and promotes tumorigenesis. J. Clin. Investig..

[45] Thulasiraman P., Kerr K., McAlister K., Hardisty S., Wistner A., McCullough I. (2019). Neuraminidase 1 regulates proliferation, apoptosis and the expression of Cadherins in mammary carcinoma cells. Mol. Cell. Biochem..

[46] Miyagi T. (2008). Aberrant expression of sialidase and cancer progression. Reviews.

[47] Chakraborty A., Dorsett K.A., Trummell H.Q., Yang E.S., Oliver P.G., Bonner J.A., Buchsbaum D.J., Bellis S.L. (2018). ST6Gal-I sialyltransferase promotes chemoresistance in pancreatic ductal adenocarcinoma by abrogating gemcitabine-mediated DNA damage. J. Biol. Chem..

[48] Chen X., Wang L., Zhao Y., Yuan S., Wu Q., Zhu X., Niang B., Wang S., Zhang J. (2016). ST6Gal-I modulates docetaxel sensitivity in human hepatocarcinoma cells via the p38 MAPK/caspase pathway. Oncotarget.

[49] Britain C.M., Dorsett K.A., Bellis S.L. (2017). The Glycosyltransferase ST6Gal-I Protects Tumor Cells against Serum Growth Factor Withdrawal by Enhancing Survival Signaling and Proliferative Potential. J. Biol. Chem..

[50] Jones R.B., Dorsett K.A., Hjelmeland A.B., Bellis S.L. (2018). The ST6Gal-I sialyltransferase protects tumor cells against hypoxia by enhancing HIF-1α signaling. J. Biol. Chem..

[51] Schultz M.J., Holdbrooks A.T., Chakraborty A., Grizzle W.E., Landen C.N., Buchsbaum D.J., Conner M.G., Arend R.C., Yoon K.J., Klug C.A. (2016). The Tumor-Associated Glycosyltransferase ST6Gal-I Regulates Stem Cell Transcription Factors and Confers a Cancer Stem Cell Phenotype. Cancer Res..

[52] Swindall A.F., Londoño-Joshi A.I., Schultz M.J., Fineberg N., Buchsbaum D.J., Bellis S.L. (2013). ST6Gal-I Protein Expression Is Upregulated in Human Epithelial Tumors and Correlates with Stem Cell Markers in Normal Tissues and Colon Cancer Cell Lines. Cancer Res..

[53] Hsieh C.-C., Shyr Y.-M., Liao W.-Y., Chen T.-H., Wang S.-E., Lu P.-C., Lin P.-Y., Chen Y.-B., Mao W.-Y., Han H.-Y. (2016). Elevation of β-galactoside α2,6-sialyltransferase 1 in a fructose-responsive manner promotes pancreatic cancer metastasis. Oncotarget.

[54] Britain C.M., Holdbrooks A.T., Anderson J.C., Willey C.D., Bellis S.L. (2018). Sialylation of EGFR by the ST6Gal-I sialyltransferase promotes EGFR activation and resistance to gefitinib-mediated cell death. J. Ovarian Res..

[55] Moore M.J., Goldstein D., Hamm J., Figer A., Hecht J.R., Gallinger S., Au H.J., Murawa P., Walde D., Wolff R.A. (2007). Erlotinib Plus Gemcitabine Compared With Gemcitabine Alone in Patients With Advanced Pancreatic Cancer: A Phase III Trial of the National Cancer Institute of Canada Clinical Trials Group. J. Clin. Oncol..

[56] Zhu Y., Srivatana U., Ullah A., Gagneja H., Berenson C.S., Lance P. (2001). Suppression of a sialyltransferase by antisense DNA reduces invasiveness of human colon cancer cells in vitro. Biochim. Biophys. Acta (BBA)-Mol. Basis Dis..

[57] Ulloa F., Real F.X. (2001). Differential Distribution of Sialic Acid in α2,3 and α2,6 Linkages in the Apical Membrane of Cultured Epithelial Cells and Tissues. J. Histochem. Cytochem..

[58] Mandal C., Tringali C., Mondal S., Anastasia L., Chandra S., Venerando B., Mandal C. (2010). Down regulation of membrane-bound Neu3 constitutes a new potential marker for childhood acute lymphoblastic leukemia and induces apoptosis suppression of neoplastic cells. Int. J. Cancer.

[59] Nath S., Mandal C., Chatterjee U., Mandal C. (2018). Association of cytosolic sialidase Neu2 with plasma membrane enhances Fas-mediated apoptosis by impairing PI3K-Akt/mTOR-mediated pathway in pancreatic cancer cells. Cell Death Dis..

[60] Pérez-Garay M., Arteta B., Llop E., Cobler L., Pagès L., Ortiz R., Ferri M.J., de Bolós C., Figueras J., de Llorens R. (2013). α2,3-Sialyltransferase ST3Gal IV promotes migration and metastasis in pancreatic adenocarcinoma cells and tends to be highly expressed in pancreatic adenocarcinoma tissues. Int. J. Biochem. Cell Biol..

[61] Momenimovahed Z., Tiznobaik A., Taheri S., Salehiniya H. (2019). Ovarian cancer in the world: Epidemiology and risk factors. Int. J. Women’s Health.

[62] Sung P.-L., Wen K.-C., Horng H.-C., Chang C.-M., Chen Y.-J., Lee W.-L., Wang P.-H. (2018). The role of α2,3-linked sialylation on clear cell type epithelial ovarian cancer. Taiwan. J. Obstet. Gynecol..

[63] Wichert B., Milde-Langosch K., Galatenko V., Schmalfeldt B., Oliveira-Ferrer L. (2018). Prognostic role of the sialyltransferase ST6GAL1 in ovarian cancer. Glycobiology.

[64] Dorsett K.A., Jones R.B., Ankenbauer K.E., Hjelmeland A.B., Bellis S.L. (2019). Sox2 promotes expression of the ST6Gal-I glycosyltransferase in ovarian cancer cells. J. Ovarian Res..

[65] Zhang X., Yang X., Chen M., Zheng S., Li J., Lin S., Wang X. (2019). ST3Gal3 confers paclitaxel-mediated chemoresistance in ovarian cancer cells by attenuating caspase-8/3 signaling. Mol. Med. Rep..

[66] Ren L.-R., Zhang L.-P., Huang S.-Y., Zhu Y.-F., Li W.-J., Fang S.-Y., Shen L., Gao Y.-L. (2015). Effects of sialidase NEU1 siRNA on proliferation, apoptosis, and invasion in human ovarian cancer. Mol. Cell. Biochem..

[67] Kvorjak M., Ahmed Y., Miller M.L., Sriram R., Coronnello C., Hashash J.G., Hartman D.J., Telmer C.A., Miskov-Zivanov N., Finn O.J. (2020). Cross-talk between Colon Cells and Macrophages Increases ST6GALNAC1 and MUC1-sTn Expression in Ulcerative Colitis and Colitis-Associated Colon Cancer. Cancer Immunol. Res..

[68] Miyazaki K., Ohmori K., Izawa M., Koike T., Kumamoto K., Furukawa K., Ando T., Kiso M., Yamaji T., Hashimoto Y. (2004). Loss of Disialyl Lewis a, the Ligand for Lymphocyte Inhibitory Receptor Sialic Acid-Binding Immunoglobulin-Like Lectin-7 (Siglec-7) Associated with Increased Sialyl Lewis a Expression on Human Colon Cancers. Cancer Res..

[69] Penrose H., Cable C., Heller S., Ungerleider N., Nakhoul H., Baddoo M., Hartono A.B., Lee S., Burow M.E., Flemington E.F. (2018). Loss of Forkhead Box O3 Facilitates Inflammatory Colon Cancer: Transcriptome Profiling of the Immune Landscape and Novel Targets. Cell. Mol. Gastroenterol. Hepatol..

[70] Wei A., Fan B., Zhao Y., Zhang H., Wang L., Yu X., Yuan Q., Yang D., Wang S. (2016). ST6Gal-I overexpression facilitates prostate cancer progression via the PI3K/Akt/GSK-3β/β-catenin signaling pathway. Oncotarget.

[71] Munkley J., Oltean S., Vodák D., Wilson B.T., Livermore K.E., Zhou Y., Star E., Floros V.I., Johannessen B., Knight B. (2015). The androgen receptor controls expression of the cancer-associated sTn antigen and cell adhesion through induction of ST6GalNAc1 in prostate cancer. Oncotarget.

[72] Haldrup C., Pedersen A.L., Øgaard N., Strand S.H., Høyer S., Borre M., Ørntoft T.F., Sørensen K.D. (2018). Biomarker potential of *ST6GALNAC3* and *ZNF660* promoter hypermethylation in prostate cancer tissue and liquid biopsies. Mol. Oncol..

[73] Tamura F., Sato Y., Hirakawa M., Yoshida M., Ono M., Osuga T., Okagawa Y., Uemura N., Arihara Y., Murase K. (2016). RNAi-mediated gene silencing of ST6GalNAc I suppresses the metastatic potential in gastric cancer cells. Gastric Cancer.

[74] Özlem Elpek G., Gelen T., Karpuzoǧlu G., Karpuzoǧlu T., Keles N. (2001). Clinicopathologic evaluation of CDw75 antigen expression in patients with gastric carcinoma. J. Pathol. A J. Pathol. Soc. Great Br. Irel..

[75] Gretschel S., Haensch W., Schlag P.M., Kemmner W. (2003). Clinical Relevance of Sialyltransferases ST6GAL-I and ST3GAL-III in Gastric Cancer. Oncology.

[76] Kawasaki Y., Ito A., Kakoi N., Shimada S., Itoh J., Mitsuzuka K., Arai Y. (2015). Ganglioside, Disialosyl Globopentaosylceramide (DSGb5), Enhances the Migration of Renal Cell Carcinoma Cells. Tohoku J. Exp. Med..

[77] Zhang Y. (2013). Epidemiology of esophageal cancer. World J. Gastroenterol..

[78] Iwaya T., Sawada G., Amano S., Kume K., Ito C., Endo F., Konosu M., Shioi Y., Akiyama Y., Takahara T. (2017). Downregulation of ST6GALNAC1 is associated with esophageal squamous cell carcinoma development. Int. J. Oncol..

[79] Yu X., Teng Y., Jiang X., Yuan H., Jiang W. (2020). Genome-Wide DNA Methylation Pattern of Cancer Stem Cells in Esophageal Cancer. Technol. Cancer Res. Treat..

[80] Mimori K., Ishii H., Inoue H., Barnard G.F., Mori M. (2008). Identification of the expression profile of apoptotic esophageal cancer cells by adenoviral-fragile histidine triad treatment. J. Gastroenterol. Hepatol..

[81] Mehta K.A., Patel K.A., Pandya S.J., Patel P.S. (2020). Aberrant sialylation plays a significant role in oral squamous cell carcinoma progression. J. Oral Pathol. Med..

[82] Shiga K., Takahashi K., Sato I., Kato K., Saijo S., Moriya S., Hosono M., Miyagi T. (2015). Upregulation of sialidaseNEU3 in head and neck squamous cell carcinoma associated with lymph node metastasis. Cancer Sci..

[83] Suzuki O., Abe M., Hashimoto Y. (2015). Sialylation by β-galactoside α-2,6-sialyltransferase and N-glycans regulate cell adhesion and invasion in human anaplastic large cell lymphoma. Int. J. Oncol..

[84] Shah I., Chou T., Tapazoglou E., Kessel D. (1984). Role of sialyltransferase in hypercupraemia of non-Hodgkin’s lymphoma. Scand. J. Haematol..

[85] Pietrobono S., Anichini G., Sala C., Manetti F., Almada L.L., Pepe S., Carr R.M., Paradise B.D., Sarkaria J.N., Davila J.I. (2020). ST3GAL1 is a target of the SOX2-GLI1 transcriptional complex and promotes melanoma metastasis through AXL. Nat. Commun..

[86] Ferreira S., Vasconcelos J., Silva R., Cavalcanti C., Bezerra C., Rêgo M., Beltrão E. (2013). Expression patterns of α2,3-Sialyltransferase I and α2,6-Sialyltransferase I in human cutaneous epithelial lesions. Eur. J. Histochem..

[87] Yu S., Wang S., Sun X., Wu Y., Zhao J., Liu J., Yang D., Jiang Y. (2021). ST8SIA1 inhibits the proliferation, migration and invasion of bladder cancer cells by blocking the JAK/STAT signaling pathway. Oncol. Lett..

[88] Torre L.A., Bray F., Siegel R.L., Ferlay J., Lortet-Tieulent J., Jemal A. (2015). Global cancer statistics, 2012. CA Cancer J. Clin..

[89] Walboomers J.M., Jacobs M.V., Manos M.M., Bosch F.X., Kummer J.A., Shah K.V., Snijders P.J., Peto J., Meijer C.J., Muñoz N. (1999). Human papillomavirus is a necessary cause of invasive cervical cancer worldwide. J. Pathol..

[90] Wu Y., Chen X., Dong W., Xu Z., Jian Y., Xu C., Zhang L., Wei A., Yu X., Wang S. (2021). ST3Gal IV Mediates the Growth and Proliferation of Cervical Cancer Cells In Vitro and In Vivo Via the Notch/p21/CDKs Pathway. Front. Oncol..

[91] Zhang X., Dong W., Zhou H., Li H., Wang N., Miao X., Jia L. (2015). α-2,8-sialyltransferase is involved in the development of multidrug resistance via PI3K/Akt pathway in human chronic myeloid leukemia. IUBMB Life.

[92] Zhou H., Li Y., Liu B., Shan Y., Zhao L., Su Z., Jia L. (2017). Downregulation of miR-224 and let-7i contribute to cell survival and chemoresistance in chronic myeloid leukemia cells by regulating ST3GAL IV expression. Gene.

[93] Yuan Q., Chen X., Han Y., Lei T., Wu Q., Yu X., Wang L., Fan Z., Wang S. (2018). Modification of α2,6-sialylation mediates the invasiveness and tumorigenicity of non-small cell lung cancer cellsin vitro and in vivo via Notch1/Hes1/MMPs pathway. Int. J. Cancer.

[94] Park J.-J., Yi J.Y., Jin Y.B., Lee Y.-J., Lee J.-S., Lee Y.-S., Ko Y.-G., Lee M. (2012). Sialylation of epidermal growth factor receptor regulates receptor activity and chemosensitivity to gefitinib in colon cancer cells. Biochem. Pharmacol..

[95] Schultz M.J., Swindall A.F., Wright J.W., Sztul E.S., Landen C.N., Bellis S.L. (2013). ST6Gal-I sialyltransferase confers cisplatin resistance in ovarian tumor cells. J. Ovarian Res..

[96] Smithson M., Irwin R., Williams G., Alexander K.L., Smythies L.E., Nearing M., McLeod M.C., Al Diffalha S., Bellis S.L., Hardiman K.M. (2022). Sialyltransferase ST6GAL-1 mediates resistance to chemoradiation in rectal cancer. J. Biol. Chem..

[97] Balmaña M., Diniz F., Feijão T., Barrias C.C., Mereiter S., Reis C.A. (2020). Analysis of the Effect of Increased α2,3-Sialylation on RTK Activation in MKN45 Gastric Cancer Spheroids Treated with Crizotinib. Int. J. Mol. Sci..

[98] Ma H., Zhou H., Song X., Shi S., Zhang J., Jia L. (2015). Modification of sialylation is associated with multidrug resistance in human acute myeloid leukemia. Oncogene.

[99] Wan H., Li Z., Cai F., Wang L. (2021). ST8SIA1 inhibition sensitizes triple negative breast cancer to chemotherapy via suppressing Wnt/β-catenin and FAK/Akt/mTOR. Clin. Transl. Oncol..

[100] Santos S.N., Junqueira M.S., Francisco G., Vilanova M., Magalhães A., Dias Baruffi M., Chammas R., Harris A.L., Reis C.A., Bernardes E.S. (2016). O-glycan sialylation alters galectin-3 subcellular localization and decreases chemotherapy sensitivity in gastric cancer. Oncotarget.

[101] Liu B., Liu Y., Zhao L., Pan Y., Shan Y., Li Y., Jia L. (2017). Upregulation of microRNA-135b and microRNA-182 promotes chemoresistance of colorectal cancer by targeting ST6GALNAC2 via PI3K/AKT pathway. Mol. Carcinog..

[102] Jia L., Luo S., Ren X., Li Y., Hu J., Liu B., Zhao L., Shan Y., Zhou H. (2017). miR-182 and miR-135b Mediate the Tumorigenesis and Invasiveness of Colorectal Cancer Cells via Targeting ST6GALNAC2 and PI3K/AKT Pathway. Am. J. Dig. Dis..

[103] Saito S., Aoki H., Ito A., Ueno S., Wada T., Mitsuzuka K., Satoh M., Arai Y., Miyagi T. (2003). Human α2,3-Sialyltransferase (ST3Gal II) Is a Stage-specific Embryonic Antigen-4 Synthase. J. Biol. Chem..

[104] Aloia A., Petrova E., Tomiuk S., Bissels U., Déas O., Saini M., Zickgraf F.M., Wagner S., Spaich S., Sütterlin M. (2015). The sialyl-glycolipid stage-specific embryonic antigen 4 marks a subpopulation of chemotherapy-resistant breast cancer cells with mesenchymal features. Breast Cancer Res..

[105] Wang X., Zhang Y., Lin H., Liu Y., Tan Y., Lin J., Gao F., Lin S. (2017). Alpha2,3-sialyltransferase III knockdown sensitized ovarian cancer cells to cisplatin-induced apoptosis. Biochem. Biophys. Res. Commun..

[106] Lillehoj E.P., Hyun S.W., Feng C., Zhang L., Liu A., Guang W., Nguyen C., Luzina I.G., Atamas S.P., Passaniti A. (2012). NEU1 Sialidase Expressed in Human Airway Epithelia Regulates Epidermal Growth Factor Receptor (EGFR) and MUC1 Protein Signaling. J. Biol. Chem..

[107] Nath S., Daneshvar K., Roy L.D., Grover P., Kidiyoor A., Mosley L., Sahraei M., Mukherjee P. (2013). MUC1 induces drug resistance in pancreatic cancer cells via upregulation of multidrug resistance genes. Oncogenesis.

[108] Lee M., Lee H.-J., Bae S., Lee Y.-S. (2008). Protein Sialylation by Sialyltransferase Involves Radiation Resistance. Mol. Cancer Res..

[109] Lee M., Lee H.-J., Seo W.D., Park K.H., Lee Y.-S. (2010). Sialylation of Integrin β1 is Involved in Radiation-Induced Adhesion and Migration in Human Colon Cancer Cells. Int. J. Radiat. Oncol..

[110] Shi M., Liu D., Duan H., Shen B., Guo N. (2010). Metastasis-related miRNAs, active players in breast cancer invasion, and metastasis. Cancer Metastasis Rev..

[111] Okazaki K., Nishigaki S., Ishizuka F., Kajihara Y., Ogawa S. (2003). Potent and specific sialyltransferase inhibitors: Imino-linked 5a′-carbadisaccharides. Org. Biomol. Chem..

[112] Schaub C., Muller B., Schmidt R.R. (1998). New sialyltransferase inhibitors based on CMP-quinic acid: Development of a new sialyltransferase assay. Glycoconj. J..

[113] Al-Saraireh Y.M.J., Sutherland M., Springett B.R., Freiberger F., Morais G.R., Loadman P.M., Errington R.J., Smith P.J., Fukuda M., Gerardy-Schahn R. (2013). Pharmacological Inhibition of polysialyltransferase ST8SiaII Modulates Tumour Cell Migration. PLoS ONE.

[114] Miyazaki T., Angata K., Seeberger P.H., Hindsgaul O., Fukuda M. (2008). CMP substitutions preferentially inhibit polysialic acid synthesis. Glycobiology.

[115] Hinou H., Sun X.-L., Ito Y. (2002). Bisubstrate-type inhibitor of sialyltransferases. Tetrahedron Lett..

[116] Hosoguchi K., Maeda T., Furukawa J.-I., Shinohara Y., Hinou H., Sekiguchi M., Togame H., Takemoto H., Kondo H., Nishimura S.-I. (2010). An Efficient Approach to the Discovery of Potent Inhibitors against Glycosyltransferases. J. Med. Chem..

[117] Wuab C.Y., Hsua C.C., Chenb S.T., Tsai Y.-C. (2001). Soyasaponin I, a Potent and Specific Sialyltransferase Inhibitor. Biochem. Biophys. Res. Commun..

[118] Chang W.-W., Yu C.-Y., Lin T.-W., Wang P.-H., Tsai Y.-C. (2006). Soyasaponin I decreases the expression of α2,3-linked sialic acid on the cell surface and suppresses the metastatic potential of B16F10 melanoma cells. Biochem. Biophys. Res. Commun..

[119] Hsu C.-C., Lin T.-W., Chang W.-W., Wu C.-Y., Lo W.-H., Wang P.-H., Tsai Y.-C. (2005). Soyasaponin-I-modified invasive behavior of cancer by changing cell surface sialic acids. Gynecol. Oncol..

[120] Iwashina T. (2003). Flavonoid Function and Activity to Plants and Other Organisms. Biol. Sci. Space.

[121] Bonfili L., Cecarini V., Amici M., Cuccioloni M., Angeletti M., Keller J.N., Eleuteri A.M. (2008). Natural polyphenols as proteasome modulators and their role as anti-cancer compounds. FEBS J..

[122] Gomes A., Fernandes E., Lima J., Mira L., Corvo M. (2008). Molecular Mechanisms of Anti-Inflammatory Activity Mediated by Flavonoids. Curr. Med. Chem..

[123] Friedman M. (2007). Overview of antibacterial, antitoxin, antiviral, and antifungal activities of tea flavonoids and teas. Mol. Nutr. Food Res..

[124] Rice-Evans C.A., Miller N.J. (1996). Biochemical Society Transactions Antioxidant activities of flavonoids as bioactive components of food. Biochem. Soc. Trans..

[125] Hidari K.I., Oyama K.-I., Ito G., Nakayama M., Inai M., Goto S., Kanai Y., Watanabe K.-I., Yoshida K., Furuta T. (2009). Identification and characterization of flavonoids as sialyltransferase inhibitors. Biochem. Biophys. Res. Commun..

[126] Li Y., Luo S., Dong W., Song X., Zhou H., Zhao L., Jia L. (2016). Alpha-2, 3-sialyltransferases regulate the multidrug resistance of chronic myeloid leukemia through miR-4701-5p targeting ST3GAL1. Lab. Investig..

[127] Severino P.F., Silva M., Carrascal M., Malagolini N., Chiricolo M., Venturi G., Forleo R.B., Astolfi A., Catera M., Videira P.A. (2018). Oxidative damage and response to Bacillus Calmette-Guérin in bladder cancer cells expressing sialyltransferase ST3GAL1. BMC Cancer.

[128] Gong A., Zhao X., Pan Y., Qi Y., Li S., Huang Y., Guo Y., Qi X., Zheng W., Jia L. (2020). LncRNA MEG3 mediates renal cell cancer progression by regulating ST3Gal1 transcription and EGFR sialylation. J. Cell Sci..

[129] Lee K.-Y., Kim H.G., Hwang M.R., Chae J.I., Yang J.M., Lee Y.C., Choo Y.K., Lee S.-S., Do S.-I. (2002). The Hexapeptide Inhibitor of Galβ1,3GalNAc-specific α2,3-Sialyltransferase as a Generic Inhibitor of Sialyltransferases. J. Biol. Chem..

[130] Lin T.-W., Chang W.-W., Chen C.-C., Tsai Y.-C. (2005). Stachybotrydial, a potent inhibitor of fucosyltransferase and sialyltransferase. Biochem. Biophys. Res. Commun..

[131] Chen J.-Y., Tang Y.-A., Huang S.-M., Juan H.-F., Wu L.-W., Sun Y.-C., Wang S.-C., Wu K.-W., Balraj G., Chang T.-T. (2011). A Novel Sialyltransferase Inhibitor Suppresses FAK/Paxillin Signaling and Cancer Angiogenesis and Metastasis Pathways. Cancer Res..

[132] Fu C.-W., Tsai H.-E., Chen W.-S., Chang T.-T., Chen C.-L., Hsiao P.-W., Li W.-S. (2021). Sialyltransferase Inhibitors Suppress Breast Cancer Metastasis. J. Med. Chem..

[133] Roa-de La Cruz L., Martínez-Morales P., Morán-Cruz I., Milflores-Flores L., Rosas-Murrieta N., González-Ramírez C., Ortiz-Mateos C., Monterrosas-Santamaría R., González-Frías C., Rodea-Ávila C. (2018). Expression analysis of ST3GAL4 transcripts in cervical cancer cells. Mol. Med. Rep..

[134] Dalangood S., Zhu Z., Ma Z., Li J., Zeng Q., Yan Y., Shen B., Yan J., Huang R. (2020). Identification of glycogene-type and validation of ST3GAL6 as a biomarker predicts clinical outcome and cancer cell invasion in urinary bladder cancer. Theranostics.

[135] Lo H.-J., Krasnova L., Dey S., Cheng T., Liu H., Tsai T.-I., Wu K.B., Wu C.-Y., Wong C.-H. (2019). Synthesis of Sialidase-Resistant Oligosaccharide and Antibody Glycoform Containing α2,6-Linked 3F^ax^-Neu5Ac. J. Am. Chem. Soc..

[136] Venturi G., Ferreira I.G., Pucci M., Ferracin M., Malagolini N., Chiricolo M., Dall’Olio F. (2019). Impact of sialyltransferase ST6GAL1 overexpression on different colon cancer cell types. Glycobiology.

[137] Duarte H.O., Rodrigues J.G., Gomes C., Hensbergen P.J., Ederveen A.L.H., de Ru A.H., Mereiter S., Polónia A., Fernandes E., Ferreira J.A. (2021). ST6Gal1 targets the ectodomain of ErbB2 in a site-specific manner and regulates gastric cancer cell sensitivity to trastuzumab. Oncogene.

[138] Britain C.M., Bhalerao N., Silva A.D., Chakraborty A., Buchsbaum D.J., Crowley M.R., Crossman D.K., Edwards Y.J., Bellis S.L. (2021). Glycosyltransferase ST6Gal-I promotes the epithelial to mesenchymal transition in pancreatic cancer cells. J. Biol. Chem..

[139] Zhang X., Pan C., Zhou L., Cai Z., Zhao S., Yu D. (2016). Knockdown of ST6Gal-I increases cisplatin sensitivity in cervical cancer cells. BMC Cancer.

[140] Minami A., Shimono Y., Mizutani K., Nobutani K., Momose K., Azuma T., Takai Y. (2013). Reduction of the ST6 β-Galactosamide α-2,6-Sialyltransferase 1 (ST6GAL1)-catalyzed Sialylation of Nectin-like Molecule 2/Cell Adhesion Molecule 1 and Enhancement of ErbB2/ErbB3 Signaling by MicroRNA-199a. J. Biol. Chem..

[141] Ozaki H., Matsuzaki H., Ando H., Kaji H., Nakanishi H., Ikehara Y., Narimatsu H. (2012). Enhancement of metastatic ability by ectopic expression of ST6GalNAcI on a gastric cancer cell line in a mouse model. Clin. Exp. Metastasis.

[142] Ferrer C.M., Reginato M.J. (2014). Sticking to Sugars at the Metastatic Site: Sialyltransferase ST6GalNAc2 Acts as a Breast Cancer Metastasis Suppressor. Cancer Discov..

[143] Chang S., He S., Qiu G., Lu J., Wang J., Liu J., Fan L., Zhao W., Che X. (2016). MicroRNA-125b promotes invasion and metastasis of gastric cancer by targeting STARD13 and NEU1. Tumor Biol..

[144] Qorri B., Harless W., Szewczuk M.R. (2020). Novel Molecular Mechanism of Aspirin and Celecoxib Targeting Mammalian Neuraminidase-1 Impedes Epidermal Growth Factor Receptor Signaling Axis and Induces Apoptosis in Pancreatic Cancer Cells. Drug Des. Dev. Ther..

[145] Zhou X., Zhai Y., Liu C., Yang G., Guo J., Li G., Sun C., Qi X., Li X., Guan F. (2020). Sialidase NEU1 suppresses progression of human bladder cancer cells by inhibiting fibronectin-integrin α5β1 interaction and Akt signaling pathway. Cell Commun. Signal..

[146] Tringali C., Lupo B., Silvestri I., Papini N., Anastasia L., Tettamanti G., Venerando B. (2012). The Plasma Membrane Sialidase NEU3 Regulates the Malignancy of Renal Carcinoma Cells by Controlling β1 Integrin Internalization and Recycling. J. Biol. Chem..

[147] Forcella M., Oldani M., Epistolio S., Freguia S., Monti E., Fusi P., Frattini M. (2017). Non-small cell lung cancer (NSCLC), EGFR downstream pathway activation and TKI targeted therapies sensitivity: Effect of the plasma membrane-associated NEU3. PLoS ONE.

[148] Lv T., Lv H., Fei J., Xie Y., Lian D., Hu J., Tang L., Shi X., Wang J., Zhang S. (2020). p53-R273H promotes cancer cell migration via upregulation of neuraminidase-1. J. Cancer.

[149] Xiao H., Woods E.C., Vukojicic P., Bertozzi C.R. (2016). Precision glycocalyx editing as a strategy for cancer immunotherapy. Proc. Natl. Acad. Sci. USA.

[150] Che J., Xu L., Gatlin W., LeBlanc R., Cao L., Broderick J., Peng L. (2022). Abstract LB221: Development of PD-L1-targeted sialidase as a novel cancer immunotherapeutic approach. Cancer Res..

[151] Cao L., Che J., Chesney A., Dixit R., Zheng N., Zane N., Broderick J., Peng L. (2022). Abstract LB203: Assessment of the safety, pharmacokinetics, and pharmacodynamics of a first-in-class cancer drug candidate E-602, a sialoglycan degrader, in non-human primates. Cancer Res..

[152] de Oliveira J.T., Santos A.L., Gomes C., Barros R., Ribeiro C., Mendes N., de Matos A.J., Vasconcelos M.H., Oliveira M.J., Reis C.A. (2015). Anti-Influenza Neuraminidase Inhibitor Oseltamivir Phosphate Induces Canine Mammary Cancer Cell Aggressiveness. PLoS ONE.

[153] Peng L., Cao L., Nerle S., LeBlanc R., Das A., Shelke S., Turner A., Che J., Siddiquee Z., Xu H. (2021). 843 Development and engineering of human sialidase for degradation of immunosuppressive sialoglycans to treat cancer. J. Immunother. Cancer.

[154] Palleon Pharmaceuticals-Press Releases (2022). Palleon Pharmaceuticals and Henlius Enter into Strategic Collaboration to Develop Bifunctional Sialidase Therapies.

[155] Guo T., Dätwyler P., Demina E., Richards M.R., Ge P., Zou C., Zheng R.B., Fougerat A., Pshezhetsky A.V., Ernst B. (2018). Selective Inhibitors of Human Neuraminidase 3. J. Med. Chem..

[156] Chen X., Varki A. (2009). Advances in the Biology and Chemistry of Sialic Acids. ACS Chem. Biol..

[157] Varki A. (2008). Sialic acids in human health and disease. Trends Mol. Med..

[158] Magesh S., Savita V., Moriya S., Suzuki T., Miyagi T., Ishida H., Kiso M. (2009). Human sialidase inhibitors: Design, synthesis, and biological evaluation of 4-acetamido-5-acylamido-2-fluoro benzoic acids. Bioorganic Med. Chem..

[159] Cairo C.W. (2014). Inhibitors of the human neuraminidase enzymes. MedChemComm.

[160] Haxho F., Neufeld R.J., Szewczuk M.R. (2016). Neuraminidase-1: A novel therapeutic target in multistage tumorigenesis. Oncotarget.

[161] ClinicalTrials.gov (2022). Glycan Mediated Immune Regulation with a Bi-Sialidase Fusion Protein (GLIMMER-01) (ClinicalTrials.gov Identifier: NCT05259696). NCT05259696.

